# The Relationship Between Past Condom Use and Condom Use Intention Among Male Construction Worker Clients of Sex Workers in the Western Cape, South Africa: A Parallel Multiple Mediator Model

**DOI:** 10.1007/s10508-025-03087-5

**Published:** 2025-02-24

**Authors:** Kamal Yakubu, Paul Bowen, Rajen Govender

**Affiliations:** 1 Nelson Mandela School of Public Governance, University of Cape Town, Private Bag X3, Rondebosch, Cape Town, 7701 South Africa; 2 Department of Construction Economics and Management, University of Cape Town, Rondebosch, Cape Town, South Africa; 3 School of Property, Construction & Project Management, RMIT University, Melbourne, Australia; 4 Institute for Social and Health Sciences, University of South Africa, Lenasia, South Africa; 5 Violence, Injury and Peace Research Unit, South African Medical Research Council, Tygerberg, South Africa

**Keywords:** Male clients of sex workers, Condom use, Reasoned Action Approach, Mediation analysis, HIV/AIDS, Sex work

## Abstract

Male clients of sex workers in South Africa are at high risk for HIV, yet limited research has examined the psychological factors influencing their condom use intentions. This study addressed this gap by assessing the mediating roles of positive attitudes towards condom use, condom use self-efficacy, and perceived norms in the relationship between past condom use and intentions to use condoms. A cross-sectional survey was used to obtain data from male construction workers who reported sexual intercourse with a sex worker in the past three months. Using a parallel multiple mediator model, the analysis revealed that condom use self-efficacy (*β* = 0.060, 95% CI [0.021, 0.107]) and positive attitudes towards condom use (*β* = 0.027, 95% CI [0.004, 0.058]) significantly mediated the relationship between past condom use and condom use intention, while perceived norms did not (*β* = − 0.001, 95% CI [− 0.007, 0.007]). These findings underscore the importance of targeting instrumental and affective attitudes and enhancing self-efficacy to promote consistent condom use in this population. Although perceived norms were not determined to be statistically significant in this study, their potential role as a mediator merits further exploration, particularly in light of the study limitations. This research highlights the need for tailored interventions to reduce HIV risk among male clients of sex workers in South Africa.

## Introduction

Condom promotion and distribution are a critical component of the South African National Strategic Plan for HIV, TB and STI Prevention (SANAC, 2023). Despite the government making male and female condoms freely available in public healthcare facilities across all districts (Beksinska et al., 2012), 56% of males and 59.1% of females aged 25–49 who indicated that they had two or more sexual partners in the past year reported that they did not use a condom at last sex in the most recent nationally representative HIV prevalence, incidence, and behaviors study (HSRC, 2023). While the sub-optimal use of condoms may in part be attributable to the increased availability of alternative HIV prevention measures in public healthcare facilities such as pre- and post- exposure prophylaxis, and the success of the universal test and treat and same day initiation of anti-retroviral treatment policies (National Department of Health of South Africa, 2017; Onoya et al., 2021)—increased and sustained investment in advocacy and behaviors-change interventions are nevertheless needed to improve condom use and reduce risky sexual behaviors, especially among individuals with concurrent or multiple sexual partners.

As a group, sex workers are likely to have concurrent or multiple sex partners, and so are at an increased risk of sexually transmitted infection (STI) and HIV infection. They are also likely to be stigmatized by society, and less likely to be reached by sexual and reproductive health interventions targeting the general population (The Aurum Institute, Anova Health Institute & UCSF, 2020; Geibel et al., 2008). Consequently, sex workers and their clients have been recognized as a “key population” to be specifically targeted with HIV/AIDS prevention interventions in the South African National Strategic Plan for HIV, TB and STI Prevention (SANAC, 2023). Key populations also include men who have sex with men (MSM), people who inject drugs, transgender persons, and incarcerated people (Shisana et al., 2015).

It is estimated that approximately 90% of sex workers in South Africa are female and that 10% identify as either male or transgender (Sonke Gender Justice, 2014). Given this gender differential, efforts to quantify HIV prevalence among female sex workers have been led by the South African National Department of Health, in collaboration with the Aurum Institute and the Anova Health Institute. Consequently, two bio-behavioral surveys referred to as the South Africa Health Monitoring Survey (SAHMS1 and SAHMS2) have been conducted using respondent-driven sampling (RDS) in the three largest metropolitan areas of South Africa: Johannesburg, Cape Town, and eThekwini (Durban). RDS is a probability-based method that relies on peer-to-peer recruitment within socially networked populations. This approach improves access to less visible members of the target population and safeguards participants’ privacy (Raifman et al., 2022).

The first survey (SAHMS1) was conducted between 2013 and 2014 (UCSF, Anova Health Institute & WRHI, 2015), and fieldwork for the second survey (SAHMS2) took place between 2017 and 2018 (The Aurum Institute, Anova Health Institute & UCSF, 2020). More recently, Kassanjee et al. (2022) have conducted a cross-sectional, nationally representative survey of female sex workers who were linked to sex worker programmes. This survey was carried out in 12 districts that were randomly selected from the 22 districts in South Africa with established sex worker programmes, ensuring representation from all nine provinces. According to this latest survey, HIV prevalence among female sex workers is estimated at 62.1% (95% CI: 60.3–65.7%).

It is important to note however, that sex workers operate within a constrained economic reality, often characterized by the threat of sexual and physical violence which affects their ability to negotiate safe sexual practices with their clients (Coetzee et al., 2017; Mukumbang, 2017). A growing body of research has sought to explain entry into, and persistence of sex work (Cunningham & Kendall, 2014; Robinson & Yeh, 2011), the cost of safe sex (Quaife et al., 2019), as well as the risk/benefit trade-offs faced by sex workers (Gertler et al., 2005; Quaife et al., 2018). In an effort to understand the transactional process of sex work and the potential risks associated with the negotiated outcome in a South African setting, George et al. (2019) examined the role of economic incentives in sex work and determined that both male and female sex workers operating at truck stops around the city of Bloemfontein, South Africa, on average, doubled their rates for condomless sex with clients, indicating that they factored in the increased risk associated with condomless sex in determining their price premium.

Furthermore, mathematical models suggest that female sex workers and their clients account for about 5–8% of heterosexual HIV transmission in South Africa (Bekker et al., 2015). Consequently, HIV prevention efforts targeting sex work have generally focused on educating and encouraging sex workers to adopt protective behaviors (Milovanovic et al., 2023). However, given the high levels of physical and sexual violence experienced by both male and female sex workers (Panday, 2013), and the absence of legal support owing to the criminalization of sex work (Human Rights Watch, 2019; Matlala & Odeku, 2021), some sex workers may not be in a position to refuse their clients’ preferences and demands.

In 2016, approximately 43% of female sex workers in Soweto, South Africa reported that they had been physically or sexually assaulted by their clients and 14% reported that they had been raped by a client (Coetzee et al., 2017). These estimates point to the fact that negotiating the terms of sexual activity with clients is a sensitive and complex issue for sex workers. According to Huschke and Coetzee (2020), one of the main barriers to consistent condom use in sex work stems from clients who threaten violence if sex workers insist on using condoms, clients who refuse to stop intercourse when the condom breaks or slips, clients who “stealth,” and clients who offer additional payment for unprotected sex (Alam & Alldred, 2021; Ebrahim, 2019). These issues are rooted in unequal gender norms that disempower sex workers and undermine consent within the sex worker-client dynamic.

As many as 35% of adolescent girls and young women in South Africa have indicated that that they have engaged in age-disparate relationships at some point (Milovanovic et al., 2023). And so, another factor influencing the sex worker-client dynamic is the prevalence of age-disparate relationships (Evans et al., 2016). Evidence suggests that adolescent girls and young women, particularly those driven by socioeconomic hardship, may engage in age-disparate relationships for material support. These relationships often perpetuate power imbalances, underpinned by patriarchal norms, which hinder women’s ability to negotiate safe sex practices (Hunter, 2002, 2005; Rutenburg et al., 2003). The overlap between adolescent girls and young women and sex work is further highlighted in empirical data which indicates that more than a third of female sex workers in the three biggest metropoles in South Africa are younger than 29 (41.6% for Cape Town; 51.8% for eThekwini (Durban) and 38.6% for Johannesburg) (The Aurum Institute, Anova Health Institute & UCSF, 2020).

Given the vulnerability of sex workers, it is not surprising that they have been the target of many interventions. Importantly, Huschke and Coetzee (2020) have argued that the focus of interventions on educating and encouraging sex workers to act responsibly is misplaced and counterintuitive, especially when viewed in the context of the ubiquity of age-disparate relationships and the threat of physical and sexual violence which they face from their clients. Consequently, they recommend that it is critical to also target the male clientele of sex workers.

A prominent reason for the relative lack of interventions specifically targeting the male clientele of sex workers in the South African context is the criminalization of sex work (Matlala & Odeku, 2021). While sex work is informally accepted—with easily identifiable street walkers and brothels in many metropoles, the continued criminality of buying and selling sex makes it difficult for researchers and interventionists to readily identify the clients of sex workers (Mgbako et al., 2017). Sex workers on the other hand, are a less challenging population to identify, study and target with interventions.

Behavior-change interventions have been central to South Africa’s HIV prevention strategies, with varying degrees of success. Government funded campaigns such as *LoveLife* and *Khomanani* have effectively increased awareness about HIV and normalized the use of condoms, particularly among younger populations (Aulette-Root, 2010; Pettifor et al., 2007). Accompanying efforts such as the implementation of medical male circumcision campaigns and education, including counselling and follow-up sessions, have also reinforced the importance of combining biomedical interventions with behavior change (Moyo et al., 2022). Peer education programs have also demonstrated efficacy in promoting safer sexual practices. For example, in secondary schools, peer-led behavioral interventions have delayed sexual debut and improved HIV knowledge among adolescents (Pettifor et al., 2005). Similar programs in workplace settings, particularly in the mining sector, have increased access to condoms and HIV testing services, showcasing the potential of targeted interventions in specific environments (Department of Mineral Resources & Energy, 2021).

However, several challenges persist. A major limitation of many behavior-change interventions is their inability to sustain behavior change over time. Pettifor et al. (2007) note that while initial knowledge gains are achieved, translating these into long-term behavioral shifts remains problematic. Structural barriers, including poverty, gender inequality, and stigma, further hinder the effectiveness of these interventions. Jewkes et al. (2010) found that economic dependence and experiences of gender-based violence often reduce women’s ability to negotiate safe sexual practices and sustain behavior change in the long term.

By focusing on condom use intentions, this study contributes to understanding the challenges of sustained condom use within a specific high-risk group: male construction workers. Findings from this research can inform the design of tailored interventions that not only address individual behavior change, but also incorporate structural factors such as workplace policies and cultural sensitivities. These insights can bridge existing gaps in HIV prevention strategies and enhance the sustainability of behavior-change efforts in similar contexts.

By way of a summary, this study is based on the a priori assumption that to effectively decrease the transmission of HIV, it is essential to include different groups of men at risk in HIV prevention and treatment interventions (Grimsrud et al., 2020), including male clients of sex workers. In pursuit of this objective, demographic data as well as behavioral and cognitive data related to condom use were collected from male clients of sex workers.

### Mobile Populations, Male Dominated Industries, and Sex Work

Mobility has been identified as a significant factor influencing HIV and STI transmission in South Africa. Research indicates that mobile populations, such as migrant laborers and individuals frequently travelling for work, are at increased risk of acquiring and transmitting HIV/STIs. This heightened vulnerability is often due to factors like limited access to healthcare services and targeted prevention interventions, social isolation, and engagement in high-risk behaviors during periods away from their regular partners. Additionally, cultural sensitivity and mistrust of health systems continue to pose significant challenges (Lurie & Williams, 2014).

Sex workers operate in a variety of environments (e.g., street corners, parks, bars, taverns, social media platforms) across South Africa’s eight metropolitan municipalities, the primary centres of commercial activity in the country (Arnott, 2006; Human Rights Watch, 2019). They are also visible in smaller towns and rural areas connected by the extensive national highway system, linking the ports on the Atlantic and Indian oceans to the interior as well as the landlocked countries in the region (Gomez et al., 2013). The vulnerability of truck drivers to HIV/AIDS has been acknowledged in the epidemiology of HIV/AIDS in Southern Africa (Marcus et al., 1995; Ramjee & Gouws, 2002) (in India, see Bhatnagar et al., 2015), and various studies have examined the role of truck drivers and sex workers in the transmission of HIV in the region (Delany-Moretlwe et al., 2014; Makhakhe et al., 2017). In addition to truck driving, the military (Mankayi, 2006), the mining industry (Campbell, 2000, 2001), and the construction industry (Harinarain & Haupt, 2014; Meintjes et al., 2007) have been identified as occupational categories with significant numbers of workers engaging in money-for-sex transactions.

The association between labour-related mobility, sex work, and the spread of HIV in Southern Africa is well documented (Correa-Agudelo et al., 2021; Decosas et al., 1995). A defining characteristic of the aforementioned occupations is that they comprise predominantly male, mobile populations (Aneke, 2015; Mangaroo-Pillay & Botha, 2020; Naysmith & Rubincam, 2012). Central to understanding these dynamics is a recognition of how masculinities shape the interactions between men in these occupations and the sex workers they engage with. Male-dominated spaces such as construction camps, mining compounds, and truck stops not only facilitate transactional sex but are also sites where notions of hegemonic masculinity are reinforced. These masculinities often valorize sexual conquest, risk-taking, and dominance while stigmatizing behaviors perceived as cautious or vulnerable (Connell & Messerschmidt, 2005; Ratele, 2013). Such gendered dynamics contribute to the normalization of risky sexual practices and heighten the vulnerability of both men and sex workers to STI’s including HIV/AIDS.

Another factor that exacerbates these risks is the legacy of apartheid-era housing arrangements for migrant laborers, which has become synonymous with overcrowding, poverty, violence and sex work (Vosloo, 2020). While truck drivers and miners have received significant research attention, construction workers, despite sharing similar structural vulnerabilities, remain understudied. Furthermore, there is little empirical work on how constructions of masculinity influence the interactions between male construction workers and the sex workers with whom they engage. This study responds to this research gap by focusing on male construction workers who reported engaging in sexual intercourse with a sex worker during the preceding three-month period.

Because of the South African construction industry’s vulnerability to HIV/AIDS, there have been several national empirical studies aimed at understanding the impact of HIV/AIDS within the sector. However, the industry’s fragmentation and the prevalence of small and medium-sized enterprises make it difficult to obtain a representative sample that allows for classic statistical generalizability (i.e. sample to population generalization). Using a convenience sample of 10,243 construction employees from 55 companies nationwide, Bowen et al. (2008) reported an HIV infection rate of 13.96% among workers in the industry. In a subsequent study, Bowen et al. (2018) expanded the convenience sample to over 57,000 construction workers and estimated an industry infection rate of 10.1%. Reflecting the national trend of better outcomes for females as compared to males, Bowen et al. (2015) found that female workers were 4.45 times (95% CI, 1.25–15.82) more likely to have undergone HIV testing compared to their male counterparts. Additionally, Bowen et al. (2017) identified gender as an indirect determinant of condom use among construction workers, with female workers more likely to indicate that they used a condom during their last sexual encounter.

Therefore, in designing HIV interventions for populations of workers in the construction industry, it is crucial to incorporate a gendered lens that addresses the role of masculinities in shaping behaviors. A behavior-change intervention refers simply to any combination of strategies designed to produce desired behavioral or health outcomes at the individual, group, or population level (Heath et al., 2015). Theories offer provisional insights into complex phenomena and so provide tentative explanations for why and under what circumstances behavior change occurs (Michie & Prestwich, 2010). Gender-sensitive interventions, informed by theoretical frameworks, are essential to addressing the structural and cultural factors that perpetuate risky sexual behaviors. Evidence from behavioral science demonstrates that theoretically informed interventions yield better outcomes (Sniehotta et al., 2014; Webster et al., 2016). Applications of behavioral science have also highlighted the importance of creating enabling environments where individuals feel empowered to make healthier decisions, particularly when such environments challenge harmful constructions of masculinity (Jewkes et al., 2015; Thaler & Sunstein, 2009).

In the context of South Africa’s HIV response, George et al. (2021) argued that sustained progress requires designing interventions that not only increase efficiency but also actively engage with the gendered power dynamics that shape health behaviors. This includes promoting alternative, equitable masculinities that support safer sexual practices and challenge the normalization of transactional sex as a marker of male identity.

### The Reasoned Action Approach

**Fig. 1:**
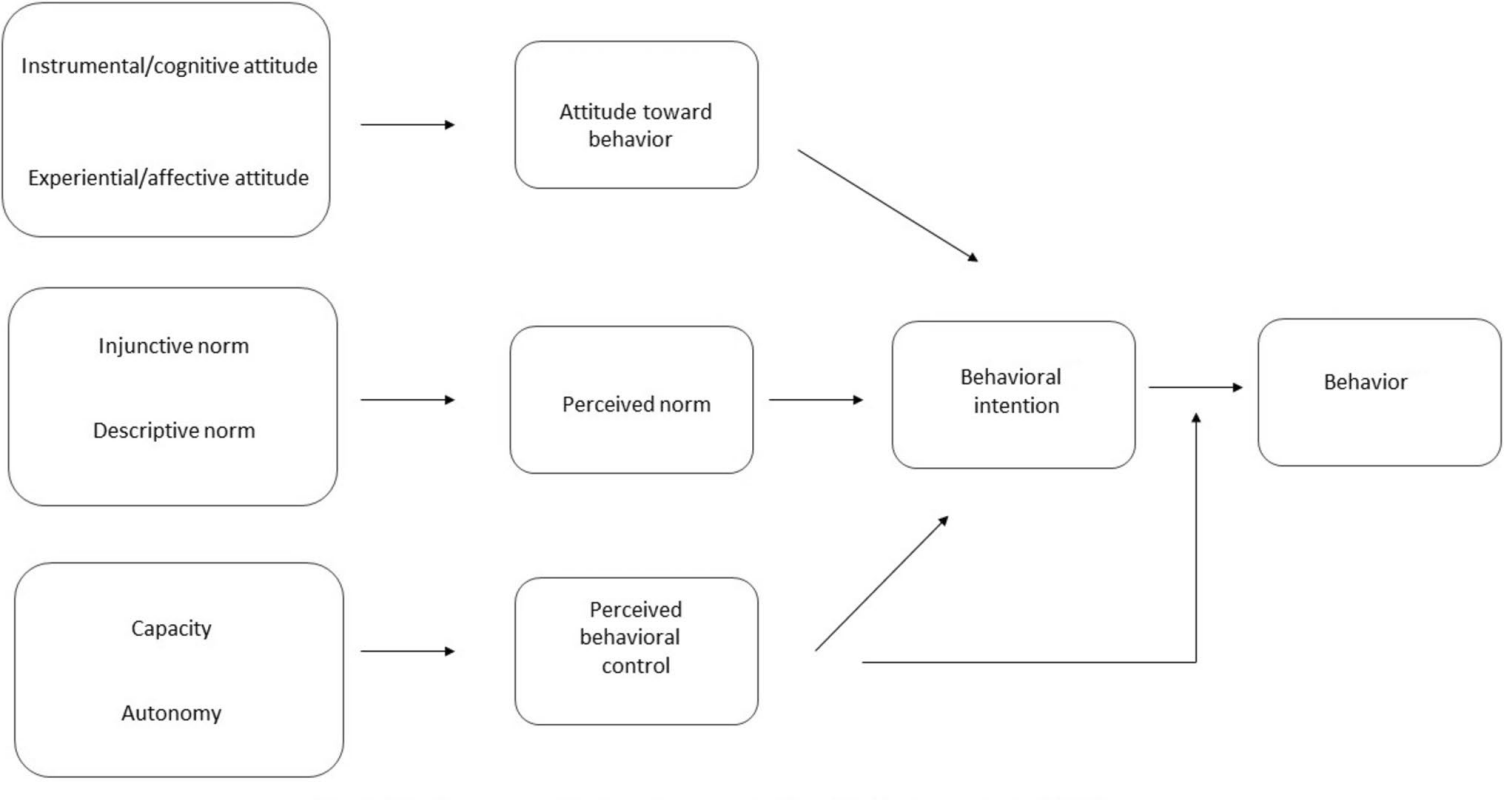
The Reasoned Action Approach (see McEachan et al., 2016)

As a contribution to the development of theory-based interventions promoting consistent condom use between sex workers and their male clients, the reasoned action approach (RAA) was used to explain the relationship between past condom use and condom use intention (Fishbein & Ajzen, 2010). The RAA is an amalgamation of the theory of reasoned action (Fishbein, 1967; Fishbein & Middlestadt, 1989) and the theory of planned behavior (Ajzen, 1985, 2002). The RAA integrates and refines these theories to provide a more nuanced understanding of how attitudes, social influences, and behavioral intentions shape behaviors (Conner et al., 2017). It posits that the most immediate determinant of a person’s behavior is their intention to perform the behavior, which is in turn influenced by three critical components: their attitude towards the behavior, perceived norms about the behavior, and perceived behavioral control, with each represented by pairs of distinct but related subcomponents (McEachan et al., 2016) (see Fig. 1).

Attitude towards behavior refers to an individual’s positive or negative evaluations of performing the behavior and is assumed to consist of cognitive/instrumental attitudes (healthy-unhealthy; valuable-worthless) and experiential/affective attitudes (pleasant-unpleasant; exciting-boring). For example, if a person believes that condoms reduce sexual pleasure, they are unlikely to have a positive attitude towards condom use. Perceived norms consist of injunctive and descriptive norms. Injunctive norms refer to the perceived social approval of others and descriptive norms refer to an individuals’ perception of what others do. For example, if a person believes that their sexual partner values consistent condom use, they may be more inclined to adopt a habit of using condoms consistently. Perceived behavioral control (henceforth PBC) reflects an individual’s perception of the ease or difficulty of performing the specified behavior. Overlaps between perceived behavioral control and self-efficacy have long been noted (Baele et al., 2001; Closson et al., 2018). Consensus, however, seems to have been reached around the idea that PBC consists of two distinct constructs—capacity and autonomy. Capacity refers to the ease or difficulty of performing a behavior, it taps into an individuals’ confidence that they can perform the behavior if they want to do so (McEachan et al., 2016). Autonomy on the other hand, “involves people’s beliefs that they have control over their behavior, and that the performance or non-performance of it is up to them” (McEachan et al., 2016, 594). By distinguishing between these two constructs, the RAA permits researchers to test for their independent effects on intention and behavior. In this study, we follow Armitage and Conner (2001) in defining capacity as self-efficacy and autonomy as perceived behavioral control.

### Current Study

**Fig. 2:**
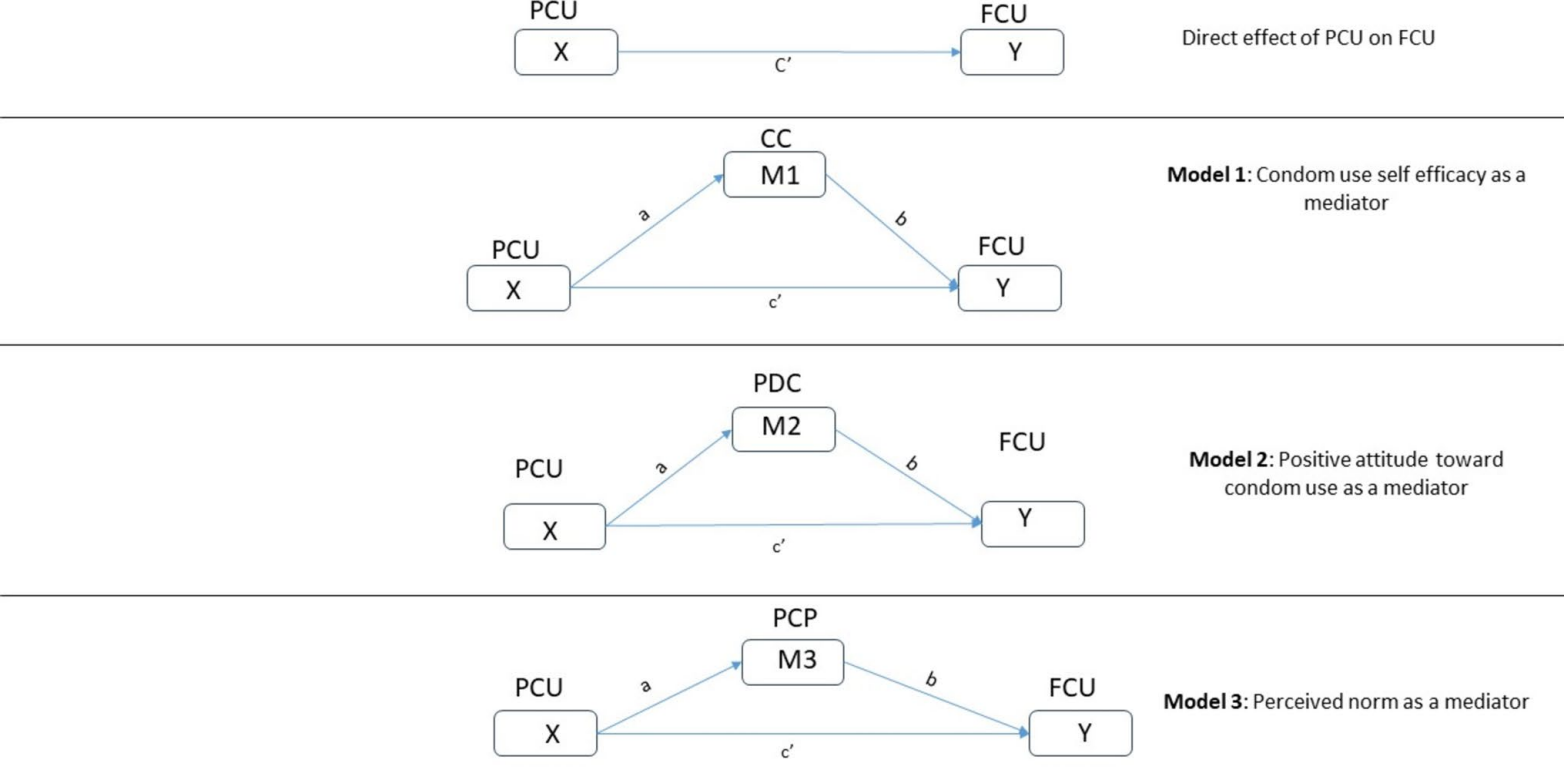
Simple mediation analyses

This study uses the RAA as a theoretical framework to examine the association between past condom use (PCU) and condom use intention (FCU) among a purposive sample of male construction worker clients of sex workers. It uses simple and parallel mediation analysis to evaluate the direct and indirect effects of perceived control over condom use (CC), positive attitude towards condom use (PDC), and perceived pressure from peers to not use a condom (PCP) (see Fig. 2). First, we hypothesize that higher levels of PCU will be associated with higher levels of FCU. Second, in line with the RAA, we hypothesize an indirect relationship between PCU and FCU via each mediator.

**Fig. 3:**
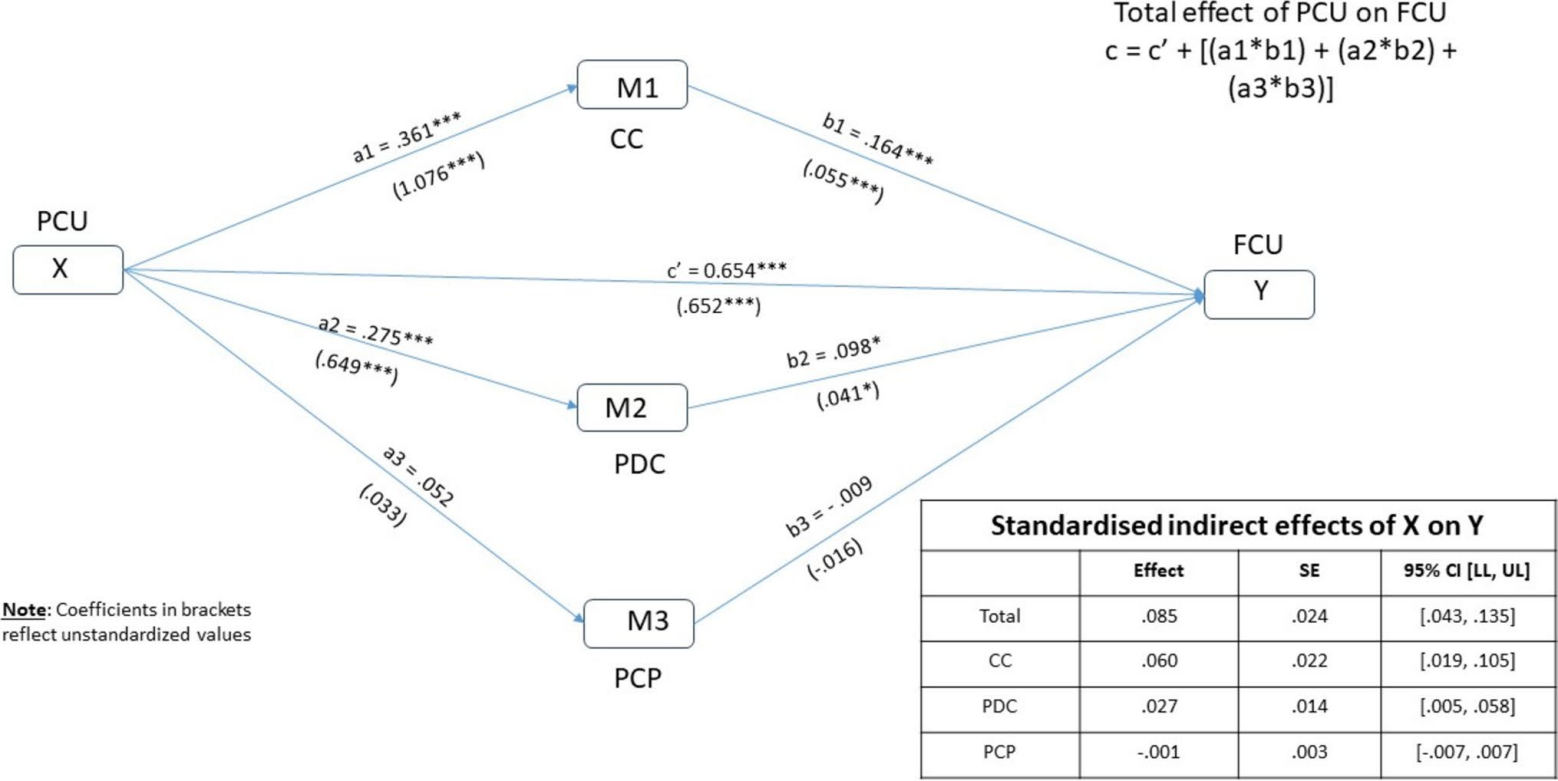
Parallel multiple mediator model

Referring to Fig. 2, the independent variable X (PCU) is associated with the dependent variable Y (FCU). Path ***c*** quantifies the total effect of X on Y. Path ***a*** represents the direct effect of X on the proposed mediator M. Path ***b*** represents the effect of M on Y, controlling for X, whereas path c′ represents the direct effect of X on Y, controlling for M. As a direct extension of the three simple mediation models, a parallel multiple mediator model was estimated (see Fig. 3). The term “parallel” refers to a relationship among the mediators in which none of the mediators directly affect each other (Cheung, 2007; Ding et al., 2014).

There are several advantages of using the parallel multiple mediator model instead of distinct simple mediation models depicted in Fig. 2. Primarily, the potential for parameter bias caused by overlooked variables is lessened when dealing with multiple hypothesized mediators. Secondly, a multiple mediator model provides researchers with the ability to ascertain the relative sizes of particular indirect effects, thereby facilitating the comparison of rival hypotheses (Preacher & Hayes, 2008).

## Method

### Participants

Data were obtained from an ongoing research project on HIV-related health behaviors among male workers in the South African construction industry (Yakubu et al., 2023), which included items on condom use and condom use intention in relation to three categories of sex partners–a regular sex partner (RSP), defined as “someone whom the participant knows very well,” a casual sex partner (CSP), defined as “someone whom the participant does not know very well,” and a sex worker (SW), defined as “someone whom the participant pays for sex” (Yakubu et al., 2023).

A convenience sampling method (Leedy & Ormrod, 2014) was employed to select construction companies and workers for data collection. The sample population consisted of all male employees present on site on the designated date and time when the participating companies had scheduled the field research visits. Data were collected across 18 construction sites spanning seven construction companies in the Western Cape province of South Africa. Furthermore, the sample included both unskilled workers and skilled tradesmen, as well as site clerks and technicians. Questionnaires were administered in three of South Africa’s 11 official languages, namely, English, Afrikaans, and *IsiXhosa*, which are the most commonly spoken languages in the Western Cape Province. Data were collected from mid-March to June 2019.

Yakubu et al. (2023) have separately examined the determinants of consistent condom use for each of RSP (*n* = 426), CSP (*n* = 287), and SW (*n* = 251). Importantly, the analysis presented in this study focuses solely on the sub-sample of 251 participants who indicated that they had sexual intercourse with a sex worker. In this regard, having had intercourse with a sex worker (in the 3-month period prior to data collection) is used as a proxy for identifying an individual as a client of a sex worker.

### Measures

#### Condom Use with Sex Workers

Past condom use and condom use intention were measured using the following two items:

1. In the last 3 months, how often did you use a condom with a sex worker?
2. The next time you have sex with a sex worker, how likely is it that you will use a condom?

In each case, the response options were: “Never”; “Rarely”; “Sometimes”; “Often”; “Always”. Scores ranged from 1 to 5.

#### Condom Use Self-Efficacy

Condom use self-efficacy was assessed using 4 items adapted from Kabikira (2010). Participants were asked the degree to which they agree or disagree with statements about condom use (e.g., “I am confident I will be able to make sure a condom is used every time I have sex.”). Response options ranged from “Strongly disagree” = 1 to “Strongly agree” = 5. Scale scores range from 4 to 20, with higher scores indicative of higher levels of confidence in using a condom (capacity) (α = 0.91).

#### Positive Attitude Towards Condom Use

A modified short-form Attitude Towards Condoms Scale (ATCS) (Roy et al., 2013) was used to assess positive attitude towards condom use. The short-form ATCS consists of 10 items structured in three dimensions: condom versus sexual satisfaction (3 items); condom versus gender dimension (3 items), and condom versus sexual interest (4 items). All three items from the condom versus sexual satisfaction dimension (e.g., “Proper use of condoms enhances sexual pleasure”) and one item from the condom versus gender dimension (“Men who use condoms show concern and responsibility to their partner(s)”) were used to measure positive attitudinal disposition towards condom use. Viewed together, these items consider the influence of both instrumental and affective attitudes. Participants were asked the degree to which they agree or disagree with the statements. The response options ranged from “strongly disagree” = 1 to “strongly agree” = 5. Scale scores range from 4 to 20, with higher scores indicating a more positive attitude towards condom use (*α* = 0.77).

#### Perceived Norm

A single item from the Condom vs. Gender Dimension sub-scale of the short-form ATCS was used to assess normative influences on condom use (Roy et al., 2013). Participants were asked the degree to which they agreed or disagreed with the following statement: “Using condoms is unmanly.”

The response options ranged from “Strongly disagree” = 1 to “Strongly agree” = 5, with higher scores indicating a higher perceived pressure from other men to not use a condom.

#### Covariates

Based on the literature review, we explored demographic, experiential and cognitive variables as covariates in the mediation analysis. Specifically, the following demographic variables—age (Stoner et al., 2019), education (Hargreaves & Glynn, 2002), marital status (Shisana et al., 2016) and employment status (De Wet-Billings & Billings, 2020); one experiential variable—having been previously diagnosed with an STI (Higgins et al., 2008); and three cognitive variables—fear of HIV infection (Gore et al., 2020), HIV/AIDS knowledge (Bowen et al., 2017), and sex-related substance use (Celio et al., 2016) were hypothesized as potential covariates.

#### Fear of HIV Infection

Five items drawn from the Perceived Risk of HIV Infection scale (PRHS) (Napper et al., 2012) were used to assess a participant’s level of fear of HIV infection (e.g., “What is your gut feeling about how likely you are to get infected with HIV?”). Response options ranged from “Extremely unlikely” = 1, “Very unlikely” = 2, “Somewhat likely” = 3, “Very likely” = 4, to “Extremely likely” = 5. The scale score range is 5–25 and higher scores indicate higher levels of fear of HIV infection (*α* = 0.68).

#### Previous STI

History of STI was measured using the following item: “Have you ever had an STI (sexually transmitted infection) excluding HIV such as: blisters or ulcers on your genitals, redness and swelling of the genitals, cloudy yellowish and greenish discharge with a smelly odor, a lot of pain when passing urine, and pain and discomfort during sexual intercourse?” Participants were asked to select one response, “No” = 0 or “Yes” = 1.

#### Risky Sex Behavior

Six items from the Sexual Risk Survey (Turchik & Garske, 2008; Turchik et al., 2010) were used to assess substance use in relation to sex (e.g., “How often have you used alcohol before or during sex?”). Response options ranged from “Never” = 1 to “Always” = 5. The scale score range is 6–30. Higher scores indicate higher levels of substance use by either the participant and/or their sex partner before or during sex (*α* = 0.86).

#### HIV/AIDS Knowledge

Five items drawn from the brief HIV Knowledge Questionnaire (HIV-KQ-18) (Carey & Schroder, 2002) were used to assess participants level of scientific knowledge concerning HIV/AIDS (e.g., “A person can get HIV by sharing a glass of water with someone who has HIV”). Response options were “True” = 0; “Don’t know” = 0; “False” = 1. The scale score range was 0–5. Higher scores indicate higher levels of scientific HIV/AIDS knowledge (*α* = 0.67). The dimensionality, reliability, and validity of the scales have been well demonstrated (see Yakubu et al., 2023, 2022).

### Statistical Analysis

The dataset was screened to detect missing, atypical, or extreme data. No outliers were identified; however, 11 participants (4.38%) had missing data for the dependent variable (i.e. condom use intention). A complete case analysis (listwise deletion) approach to missing data was adopted, and so any observation that had a missing value was excluded. Consequently, the sample size was reduced from *n* = 251 to *n* = 240. The normality of the relevant measures was assessed using the Kolmogorov–Smirnov test, the Shapiro–Wilk test, a histogram, and Q-Q plots (Table 1).

**Table 1:** Demographics, measures, covariates, sample items, and response options

Items and constructs	Sample items	No. of items	Response options and point of scales
*Condom use with sex workers*			
In the last 3 months, how often did you use a condom with a sex worker? [Q38d]	–	–	“Never” = 1 to “Always” = 5
The next time you have sex with a sex worker, how likely is it that you will use a condom? [Q39d]	–	–	“Never” = 1 to “Always” = 5
*Demographic characteristics*			
Relationship status [Q3]	–	–	Divorced, separated, widowed, or never married = 0; Married/living with a partner = 1
Age [Q1]	–	–	18–34 = 1; 35 and over = 2
Education [Q6]	–	–	Primary or less = 1; Secondary exposed to/completed = 2; Tertiary exposed to/completed = 3
Employment status [Q8]	–	–	Casual or contract = 1; Permanent = 2
*Covariates*			
Fear of HIV infection (Scale score range: 5–25); α = .68	“Getting HIV is something that I am concerned about” [Q48]	5	“Extremely unlikely” = 1, to “Extremely likely” = 5
Previous STI	“Have you ever had an STI excluding HIV such as blisters or ulcers on your genitals …?” [Q60]	1	“No” = 0; “Yes” = 1
Risky Sex Behavior (Scale score range: 6–30); α = .85	“How often has your partner used drugs before or during sex?”	6	Never’ = 1 to “Always” = 5. “Not applicable” = 6
HIV/AIDS knowledge (Scale score range: 0–5); α = .67	“A person can get HIV by touching someone who has HIV” [Q32a]	5	“True” = 0; “Don’t know’ = 0; “False” = 1
*Mediators*			
Control over condom use (Scale score range: 4–20); α = .91	“I am confident I will be able to make sure a condom is used every time I have sex” [Q40b]	4	“Strongly disagree” = 0 to “Strongly agree” = 5
Positive attitude towards condom use (Scale score range: 4–20); α = .77	“Condoms are the best way to protect myself from HIV” [Q36i]	4	“Strongly disagree” = 1 to “Strongly agree” = 5
Perceived norm	“Using condoms is unmanly” [Q36h]	1	“Strongly disagree” = 1 to “Strongly agree” = 5

Question numbers in the questionnaire are given in brackets

The study variables were determined not to be normally distributed, resulting in the use of non-parametric tests to investigate statistical relationships. Three sets of analyses were performed. First, bivariate analyses were used to determine whether the study variables were significantly associated. Specifically, the Mann–Whitney U test was used to assess the relationship between the five measures (PCU, FCU, CC, PDC, PCP) and the categorical variables (Table 2) and the Spearman’s rank correlation coefficient was used to examine the association with the continuous variables (Table 3). A variable was only included as a covariate in the mediation analysis if it was found to be significantly associated with one or more of PCU, FCU, CC, PDC, and PCP.

**Table 2:** Descriptive statistics and Mann–Whitney U test (*n* = 240)

Construct	Total	%	PCU	FCU	CC	PDC	PCP
*Relationship status*			.696	.860	.459	.406	.922
Single, widowed, separated or divorced	134	55.8					
Married or living with a partner	106	44.2					
*Education*^*a*^			.413	.203	.021	.021	.043
Primary or less	53	22.1					
Exposed to or completed (Secondary)	170	70.8					
Exposed to or completed (Tertiary)	17	7.1					
*Age*			.210	.328	.806	.354	.610
18–34 years	130	54.2					
35 and over	110	45.8					
*Employment status*			.806	.966	.684	.237	.982
Casual or Contract	140	58.3					
Permanent	100	41.7					
*Previous STI*			.066	.083	.012	.036	.437
No	206	85.8					
Yes	34	14.2					

Use of a condom with a sex worker within last 3 months (PCU); Intention to use a condom at next sex with a sex worker (FCU); Control over condom use (CC); Positive attitude to condom use (PDC); perceived norm (PCP)

^a^Kruskal–Wallis H Test

**Table 3:** Descriptive statistics and Spearman’s rank correlation analysis (*n* = 240)

Construct	PCU	CC	PDC	PCP	FCU	FHIV	RSB	HAK
Use of a condom with a sex worker within last 3 months (PCU)	–							
Control over condom use (CC)	.38***	–						
Positive attitude to condom use (PDC)	.27***	.45***	–					
Perceived norm (PCP)	.04	.04	.04	–				
Intention to use a condom at next sex with a sex worker (FCU)	.76***	.47***	.34***	.04	–			
Fear of HIV infection (FHIV)	.19**	.16*	.15*	− .06	.11	–		
Risky sex behavior (RSB)	.18**	.03	.01	.13*	.13*	.21***	–	
HIV/AIDS knowledge (HAK)	.18**	.16*	.13*	− .14*	.27***	− .03	− .20**	–
Mean	2.93	12.98	13.58	2.06	3.05	11.57	9.10	3.45
SD	1.77	5.28	4.18	1.12	1.76	4.48	4.25	1.40

^*^p < 0.05; **p < 0.01; ***p < 0.001

The SPSS macro, PROCESS version 4.2 (Hayes, 2022) was used to test CC, PDC, and PCP as potential mediators of the relationship between PCU and FCU. PROCESS employs non-parametric bootstrapping to examine the direct and indirect effects of mediators on the relationship between independent and dependent variables. Bootstrapping involves repeatedly sampling from the data with replacement to create an approximation of the sampling distribution and to generate confidence intervals. In this study, 5000 bootstrap resamples were used to generate 95% confidence intervals. Mediation was deemed to be statistically significant if the 95% CI bias-corrected lower and upper limits for the indirect effects did not include zero (Hayes & Scharkow, 2013). Three distinct simple mediator models, one with each mediator: (1) CC; (2) PDC; and (3) PCP were initially tested, after which a parallel multiple mediator model was tested using template 4 of the PROCESS macro (Hayes, 2022). All models were examined twice, with and without covariates (Ledgerwood, 2019; Wang et al., 2017). SPSS for Windows (ver. 29) was used for descriptive statistics and bivariate analyses (IBM Corporation, 2024).

## Results

### Demographic Characteristics

The participants were all males, aged between 18 and 60 years. Approximately 58% of the participants were casual or contract workers, and 56% described their relationship status as single, widowed, separated, or divorced. Nearly 70% had completed or been exposed to secondary school level education, while 7% had tertiary level education.

### Bivariate Analyses

A Kruskal–Wallis H test showed that there was a statistically significant difference in CC, PDC, and PCP scores across the three education categories, and participants who had higher levels of education recorded higher scores for CC and PDC and lower scores for PCP. Specifically, participants who had completed or been exposed to tertiary education (Md = 16) and those who had completed or been exposed to secondary education (Md = 15) recorded a higher median score than those who had completed or been exposed to primary education (Md = 11). This pattern was consistent for PDC as well, and participants who had completed or been exposed to primary education recorded a lower median score (Md = 12) than the other two groups.

For PCP, participants who had completed or been exposed to tertiary education recorded a lower median score (Md = 1) than the other two groups (which both recorded Md = 3).

Aside from education, none of the sociodemographic variables were found to be significantly associated with either PCU, FCU, CC, PDC or PCP. Given this finding, education was included in the mediation models as a statistical control variable.

No significant difference in the scores of PCU, FCU and PCP across the categories of previous STI were observed. A Mann–Whitney U test revealed a significant difference in CC scores between participants who had not been previously diagnosed with an STI (M = 115.96, *n* = 206) and those who had been previously diagnosed with an STI (M = 148.03, *n* = 34), U = 4438, z = 2.51, *p* = 0.012, *r* = 0.16. There was also a statistically significant difference in PDC scores between participants who had not been previously diagnosed with an STI (M = 116.71, *n* = 206) and those who had been previously diagnosed with an STI (M = 143.49, *n* = 34), U = 4283.50, z = 2.10, *p* = 0.036, *r* = 0.13. Given this finding, previous STI was included in the mediation models as a statistical control variable.

Table 3 depicts the correlations among the main study variables and the rest of the hypothesized covariates along with their means and standard deviations. Spearman’s rank correlation coefficient showed that PCU was positively and significantly associated with FCU (*r* = 0.76, *p* < 0.001) as expected. Furthermore, aside from PCP (*r* = 0.04, *p* = 0.517), PCU was determined to be significantly associated with all the mediators and hypothesized covariates. Similarly, FCU was determined to be significantly associated with all the mediators and hypothesized covariates except for PCP (*r* = 0.04, *p* = 0.540) and FHIV (*r* = 0.11, *p* = 0.090). Given that each of FHIV, RSB and HAK were found to be statistically associated with one or more study variables, all three were retained and used as statistical control variables in the mediation models.

### Testing the Simple Mediation Models

Three simple mediation models were specified and tested, one for each mediator (Fig. 2). Standardized direct and indirect effects are presented in Table 4. The 95% CI bias-corrected lower and upper limits for the indirect effects in the CC and PDC models did not include zero, and so it was concluded that CC and PDC mediate the relationship between PCU and FCU. On the other hand, the 95% CI bias-corrected lower and upper limits of the indirect effect of PCP included zero, and so it was concluded that PCP did not mediate the relationship between PCU and FCU. Specifically, when CC and PDC were utilized as simple mediators, both the direct and indirect effects were statistically significant. However, when PCP was employed in a simple mediation model, only the direct effect between PCU and FCU was determined to be statistically significant (*β* = 0.738, *p* < 0.001).

**Table 4:** Simple mediation models and the effects of CC (M_1_), PDC (M_2_), and PCP (M_3_) on the relationship between PCU (X) and FCU (Y)—with and without covariates

	Variable X		Variable Y	Standardized effects			
Model (with covariates)	PCU	Variable M	FCU	Effect of X on M	Effect of M on Y	Direct effect	Indirect effect
				*a*	*b*	*c*	(a*b)
				95% CI [LL, UL]	95% CI [LL, UL]	95% CI [LL, UL]	95% CI [LL, UL]
1	*Past condom use*	CC (M_1_)	*Condom use Intention*	.360***	.205***	.665***	0.074
				[.713, 1.439]	[.039, .097]	[.576, .750]	[.035, .122]
2		PDC (M_2_)		.275***	.160***	.695***	.044
				[.349, .948]	[.032, .1035]	[.607, .779]	[.018, .081]
3		PCP (M_3_)		.052	.018	.738***	.001
				[− .052, .119]	[− .101, .157]	[.651, .821]	[− .005, .010]
Model (without covariates)							
1		CC (M_1_)		.407***	.212***	.681***	.086
				[.866, 1.563]	[.042, .099]	[.593, .764]	[.042, .136]
2		PDC (M_2_)		.315***	.169***	.714***	.053
				[.457, 1.028]	[.036, .107]	[.628, .795]	[.024, .090]
3		PCP (M_3_)		.031	.004	.767***	.001
				[− .062, .101]	[− .123, .134]	[.683, .846]	[− .006, .007]

Use of a condom with a sex worker within last 3 months (PCU); Intention to use a condom at next sex with a sex worker (FCU); Control over condom use (CC); Positive attitude to condom use (PDC); Perceived norm (PCP)

Only Standardized effects are presented. *p < 0.05; **p < 0.01; ***p < 0.001

The results reported above include the covariates. The results of the mediation analysis without covariates are also shown in Table 4. There was no substantive difference between the simple mediation models with covariates and without.

### Testing the Parallel Multiple Mediator Model

Given that the results of the simple mediation analyses were not substantively different with or without covariates, the parallel multiple mediator model was conducted with covariates. Table 5 reports on the standardized and unstandardized effects, and Fig. 3 provides a visual summary of the results.

**Table 5:** Parallel multiple mediator model and the standardized and unstandardized effects of CC (M_1_), PDC (M_2_) and PCP (M_3_) on the relationship between PCU (X) and FCU (Y)

Effect of X on M_1_	Effect of X on M_2_	Effect of X on M_3_	Effect of M_1_ on Y	Effect of M_2_ on Y	Effect of M_3_ on Y	Direct effect c’ (X–Y)	Indirect effect (a*b) 95%CI M on Y	Total Indirect effect 95%CI M on Y	Total effect c = c’ * (a*b) X on Y
Standardized effects									
.361***	.275***	.052	.164***	.098*	− .009	.654***	**M**_**1**_	.085	.737***
[.713, 1.440]	[.349, .948]	[− .052, 1.185]	[.023, .086]	[.003, .080]	[− .140, .109]	[.564, .739]	.060	[.043, .135]	[.652, .822]
							[.021, .107]		
							**M**_**2**_		
							.027		
							[.004, .058]		
							**M**_**3**_		
							− .001		
							[− .007. .007]		
Unstandardized effects									
1.076***	.649***	.033	.055***	.041*	− .016	.652***	**M**_**1**_	.085	.737***
[.713, 1.440]	[.349, .948]	[− .052, .119]	[.023.086]	[.003, .080]	[− .140, .109]	[.564, .739]	.059	[.042, .134]	[.652, .822]
							[.019, .106]		
							**M**_**2**_		
							.027		
							[.004, .060]		
							**M**_**3**_		
							− .001		
							[− .007, .007]		

Use of a condom with a sex worker within last 3 months (PCU); Intention to use a condom at next sex with a sex worker (FCU); Control over condom use (CC); Positive attitude to condom use (PDC); Perceived norm (PCP)

*p < 0.05; **p < 0.01; ***p < 0.001

Controlling for the covariates and for the other mediators, the total indirect effect of the model was determined to be statistically significant (IE_overall_ = 0.085 [95% CI = 0.043, 0.135]). The only hypothesized mediator that was not found to have a unique indirect effect was PCP (*β* = − 0.001 [95% CI = − 0.007, 0.007]). Statistically significant unique indirect effects were exhibited by CC (*β* = 0.060 [95% CI = 0.021, 0.107]) and PDC (*β* = 0.027 [95% CI = [0.004, 0.058]). In terms of magnitude, the direct effect between PCU and FCU (β = 0.654, *p* < 0.001) was determined to be the strongest followed by CC (*β* = 0.164, *p* < 0.001*)* and PDC (*β* = 0.098, *p* < 0.05). PCU was significantly associated with CC (*β* = 0.361, *p* < 0.001*)*, as well as PDC (*β* = 0.275, *p* < 0.001), but not PCP (*β* = 0.052, *p* = 0.444).

## Discussion

HIV prevention efforts that focus solely on sex workers overlook a critical aspect: the male clientele (Huschke & Coetzee, 2020). In the heterosexual transmission of STIs in general and HIV in particular, male clients of sex workers act as a bridging population, linking high-risk groups (e.g., sex workers) to low-risk groups (e.g., wives, girlfriends, and other sexual partners) (Morse-Karzen, 2020). Moreover, negotiations for safe sex occurs in a constrained environment where male clients often hold more power than sex workers (Coetzee et al., 2017; Morrell et al., 2012).

Given the historical association between migrant male populations, worker hostels, and sex workers in South Africa (Vosloo, 2020), this study aimed to better understand condom use among men working in construction—a male-dominated industry with a significant mobile population. Using the RAA as a theoretical framework, the study examined condom use intentions among male construction workers who reported engaging in sexual intercourse with a sex worker in the three-month period leading up to the survey. Specifically, the study tested the mediation roles of positive attitudes towards condom use, condom use self-efficacy, and perceived norms in the association between past condom use and future condom use intention.

Consistent with the RAA, greater intention to use a condom at the next sexual encounter was directly associated with past condom use, condom use self-efficacy, and positive attitudes towards condom use. Perceived norms, however, were not directly associated with condom use intentions. The weak predictive power of the normative component of the RAA has been noted in previous studies (Armitage & Conner, 2001; Fishbein & Ajzen, 2010; McEachan et al., 2016). Nonetheless, in this study, perceived norm was measured using a single questionnaire item, and its weak predictive power may be partly attributable to limitations in measurement. These findings suggest that high levels of condom use self-efficacy and positive attitudes towards condom use are key drivers of greater condom use intentions.

Past condom use was found to be indirectly associated with condom use intention via self-efficacy and positive attitudes towards condom use. It was not indirectly associated with perceived norms. The inclusion of both instrumental and affective attitudes in the measure of positive attitude towards condom use highlights that male construction worker clients of sex workers may be more likely to intend to use condoms if they perceive them as valuable or as enhancing sexual pleasure. Similarly, the link between self-efficacy and condom use intention suggests that men confident in their ability to use condoms are more likely to form intentions to use them. Based on these findings, interventions aiming to promote consistent condom use among male clients of sex workers should prioritize enhancing positive attitudes (both instrumental and affective) and strengthening confidence and control beliefs related to condom use.

### Implications

This study has implications for (1) developing interventions to promote consistent condom use among male clients of sex workers, and (2) advancing our understanding of the individual-level determinants of condom use intentions. Additionally, it contributes to the growing body of evidence underscoring the relevance of behavioral science in understanding HIV-related health behaviors and informing intervention development in the South African context (George et al., 2021).

Contrary to traditional assumptions that focus primarily on risk reduction, this study aligns with evidence-based interventions that take pleasure and sexual satisfaction into account as key motivators of condom use. Although pleasure and minimized sexual satisfaction are often cited as barriers to consistent condom use, masculinity-focused HIV interventions in South Africa have recognized the predominance of male pleasure as a driving force in sexual behavior (Mankayi, 2006; Mfecane, 2008). These interventions emphasize the influence of male sexual scripts—which link masculinity with sexual dominance and pleasure—on risky behaviors associated with HIV transmission (Kaufman et al., 2014). Thöle-Muir (2014) for instance, has provided valuable insights into how masculinity-focused interventions can shift harmful gender norms and reduce risky behaviors associated with HIV transmission. The findings emphasize that entrenched norms, such as the entitlement to multiple partners and inconsistent condom use, remain barriers to HIV prevention in South Africa.

Given that this study’s measure of positive attitudes towards condom use incorporated both affective and instrumental dimensions, the findings suggest that interventions promoting consistent condom use should adopt a sex-positive approach. Such an approach emphasizes the integration of pleasure and sexual satisfaction as core components of condom use promotion. However, interventions must extend beyond sex positivity to address the broader implications of entrenched masculine sexual scripts. For instance, the normalization of male pleasure and dominance within heterosexual interactions can reinforce power imbalances, undermine safe sex practices, and contribute to the spread of STIs. Initiatives like the Tavern Intervention Programme (TIP) illustrate how challenging harmful masculinity ideals can drive positive change. By fostering alternative masculinities that emphasize respect, mutual decision-making, and safe sexual practices, TIP demonstrates the importance of directly engaging with masculine sexual norms to mitigate risky behaviors such as unprotected sex and engagement with multiple partners (Thöle-Muir (2014).

Sex positivity, as defined by Burnes et al. (2017) and Cruz et al. (2017), is a non-pathologizing approach to sexuality that recognizes sexual activity as developmentally normative and integral to psychological health, relational satisfaction, and overall quality of life. While this framework is valuable, it must be paired with strategies that critically examine masculinity and challenge deeply ingrained sexual scripts. Such interventions should aim to address the intersection of hegemonic masculinity and sexual risk-taking, while promoting alternative, health-affirming masculinities.

To be effective, intervention strategies must be context-sensitive, taking into account cultural and industry-specific variations in beliefs, attitudes, and norms (Galea & Chappell, 2022). These strategies should also be implemented at multiple levels—individual, relational, and structural—to maximize their impact in promoting consistent condom use among male clients of sex workers.

### Limitations and Suggestions for Future Research

Several limitations need to be considered in interpreting the results of the study. First, because of the convenience sampling approach adopted the sample is not representative of all male construction worker clients of sex workers and so, future research should usefully employ probability sampling techniques to increase statistical generalizability. Second, due to the cross-sectional nature of the data, no causal conclusions can be drawn from the mediation effects illustrated. Future research should make use of post-hoc reverse mediations (Munn & James, 2022) or adopt longitudinal designs to examine prospective relationships. While emergent literature suggests that the use of self-report data in sexual behavior research presents no major problems (DiClemente et al., 2013; Goldberg et al., 2014), the exclusive reliance on retrospective self-reports from participants may have nevertheless had an influence on the analysis (Strassberg & Lowe, 1995).

An additional limitation pertains to the study’s assessment of perceived norms, which were measured using a single item from the condom vs. gender dimension sub-scale of the short-form ATCS. While this measure provides important insights, it may not adequately capture the complexity of gender norms and their influence on condom use behaviors. Future studies would benefit from integrating the Gender Equitable Men (GEM) scale, a validated tool that assesses attitudes towards gender norms and masculinity (Pulerwitz & Barker, 2008). The GEM scale has been shown to reveal nuanced relationships between gender attitudes and health behaviors, offering a more comprehensive framework for understanding how masculinity-related norms influence HIV-related behaviors such as the solicitation of sex and condom use. Incorporating the GEM scale could also address the study’s inability to fully explore the role of gender dynamics, particularly in the context of male-dominated occupational environments like construction work. This would allow future research to identify actionable points for interventions targeting gender inequities and harmful masculinities.

Importantly, the study did not include an assessment of the gender of the sex worker. As such, we were unable to determine whether participants engaged in sexual transactions with female sex workers, male sex workers, or both. This limitation means the findings do not account for the potential HIV risks associated with same-sex transactional sex. Emerging evidence suggests that men in traditionally hypermasculine occupational settings, such as construction, may not exclusively engage in heterosexual transactional sex (Sandfort et al., 2008). Future studies should explicitly assess the gender of sex workers to better understand the dynamics of HIV risk in this at-risk population group and develop more nuanced interventions.

Although we controlled for several covariates (education, previous STI, fear of HIV infection, risky sex behavior, and HIV/AIDS knowledge), we recognize that there are other potential predictors and pathways through which condom use intention may be linked to past condom use. Thus, more research examining alternative mediating pathways is warranted. Relatedly, the use of the RAA in this study provides valuable insights into the cognitive and behavioral determinants of HIV-related health behaviors (Fishbein & Ajzen, 2010). However, it is important to acknowledge its limitations, particularly in the South African context, where HIV vulnerability is deeply embedded in structural, racialized, and gendered dynamics. One significant critique of RAA is its overemphasis on rationality, assuming that individuals make decisions through deliberate reasoning, which may overlook the role of emotions, unconscious motivations, and spontaneous actions. Additionally, while the framework accounts for subjective norms, it has been critiqued for insufficiently addressing broader social, cultural, and structural influences, which are crucial in shaping health behaviors in contexts like South Africa. Furthermore, the RAA’s limited consideration of habitual or impulsive behaviors constrains its applicability to contexts where such actions significantly contribute to health outcomes.

To address these limitations, alternative frameworks such as gender-transformative approaches and structural violence paradigms offer promising avenues for research and intervention. Gender-transformative interventions aim to challenge and change harmful gender norms that perpetuate HIV risk. For example, the Stepping Stones and Creating Futures intervention in South African informal urban settlements combined gender-transformative strategies with economic strengthening, resulting in shifts in gender norms, reductions in intimate partner violence (IPV), and lower HIV vulnerability among young people (Gibbs et al., 2020). Similarly, addressing structural violence and systemic social structures that harm or disadvantage individuals, has proven essential in the South African healthcare context. Discriminatory attitudes from healthcare workers, a form of structural violence, often impede adolescents’ and young people’s access to sexual and reproductive health services. Initiatives like men-only clinics have been developed to address these barriers. These clinics provide male-friendly services and create a more welcoming environment, leading to increased HIV testing and treatment uptake among men (Cassidy et al., 2023). By integrating these perspectives, future research can provide a more comprehensive understanding of the multifaceted factors contributing to HIV risk and prevention behaviors. This would enable the design of interventions that address both individual and structural determinants, ultimately enhancing the effectiveness of HIV prevention efforts.

### Conclusion

This study investigated the mediating effects of past condom use via condom use self-efficacy, positive attitude towards condom use and perceived norm on condom use intention in serial and in parallel. Results show that condom use self-efficacy, positive attitude towards condom use (but not perceived norm) mediate the effects of past behavior on condom use intention. The study addresses a crucial gap in the HIV response through its focus on condom use intention of male clients of sex workers. Furthermore, the results extend existing research by illustrating how condom use intention can be explained by the RAA. Although the measure for perceived norm was not determined to be a mediator, the association between condom use intention, past behavior and perceived norm requires further investigation given the limitations of the present study.

## Data Availability

Due to the sensitive nature of the research and the associated ethical restrictions, the supporting data are not available.
